# Day of Surgery Admission in Total Joint Arthroplasty: Why Are Surgeries Cancelled? An Analysis of 3195 Planned Procedures and 114 Cancellations

**DOI:** 10.1155/2016/1424193

**Published:** 2016-11-16

**Authors:** David M. Dalton, Enda G. Kelly, Terence P. Murphy, Gerry F. McCoy, Aaron A. Glynn

**Affiliations:** ^1^University Hospital Limerick, Limerick, Ireland; ^2^Cappagh National Orthopaedic Hospital, Dublin, Ireland; ^3^Sunnybrook Hospital, Toronto, ON, Canada; ^4^Kilcreene Orthopaedic Hospital, Kilkenny, Ireland; ^5^Our Lady of Lourdes Hospital, Drogheda, Ireland

## Abstract

*Background*. Day of surgery admission (DOSA) is becoming standard practice as a means of reducing cost in total joint arthroplasty.* Aims*. The aim of our study was to audit the use of DOSA in a specialty hospital and identify reasons for cancellation.* Methods*. A retrospective study of patients presenting for hip or knee arthroplasty between 2008 and 2013 was performed. All patients were assessed at the preoperative assessment clinic (PAC).* Results*. Of 3195 patients deemed fit for surgery, 114 patients (3.5%) had their surgery cancelled. Ninety-two cancellations (80%) were due to the patient being deemed medically unsuitable for surgery by the anaesthetist. Cardiac disease was the most common reason for cancellation (*n* = 27), followed by pulmonary disease (*n* = 22). 77 patients (67.5%) had their operation rescheduled and successfully performed in our institution at a later date.* Conclusion*. DOSA is associated with a low rate of cancellations on the day of surgery. Patients with cardiorespiratory comorbidities are at greatest risk of cancellation.

## 1. Background and Aims

Hip and knee arthroplasty are safe, cost effective procedures [1]. The number of these procedures being performed is rising and this trend is projected to continue over the next 20 years [2, 3]. The increase in demand for these operations represents a significant financial burden on the health services. The average Medicare A reimbursement per case of a primary hip or knee arthroplasty is $9,484 [4]. With increased financial scrutiny of elective surgery, a number of papers have been published analyzing costs and means of reducing these [5–7].

Day of surgery admission (DOSA) has been widely adopted as a means of reducing average length of hospital stay and costs associated with surgery. It has been successfully used in hip and knee arthroplasty [8] and in 2009 was endorsed by the Health Service Executive in Ireland as official policy with a target cancellation rate of less than 5% for patients attending for surgery [9].

For a patient to be considered for DOSA, an effective preoperative evaluation must be carried out and comorbidities optimized to the satisfaction of the anesthesiologist and surgeon managing the patients' care on the day of surgery. A deficit in this process has the potential to lead to a patient having their procedure cancelled, with subsequent loss of operating room time.

The aim of the current study is to audit DOSA in our unit and identify causes of cancelled surgical procedures.

## 2. Methods

Institutional Review Board approval was sought for this study. A retrospective review of patients presenting to our unit for primary lower limb arthroplasty, over a six-year period between January 1st 2008 and December 31st 2013, was performed. Ours is a “stand-alone” specialty unit without intensive care capability, which performs procedures on low-to-intermediate risk patients (American Society of Anesthesiologists (ASA) Score 1–3) [10]. Just over 50% of these patients do not have health insurance cover. Seven attending orthopedic surgeons worked in the facility during the time under consideration.

All patients scheduled for total hip and total knee arthroplasty were assessed at a preoperative assessment clinic (PAC), which is led by attending physician. A nurse and an orthopedic resident also assess these patients. A directed history is taken to identify any chronic conditions that require specialist investigation and optimization preoperatively.

Patients are again seen by an orthopedic resident one week prior to their procedure to ensure these investigations have been performed, to obtain consent for surgery, and to take a blood sample for type and screening.

Patients who underwent lower limb joint arthroplasty were identified from the in-hospital Health Information Patient Enquiry (HIPE) system. A detailed chart review of any patients surgeries cancelled on the day of surgery was performed. Records from their hospital admission as well as from PAC were analyzed. All haematological, radiological, and relevant cardiac investigations including electrocardiographs, echocardiographs, chest X-rays, and blood counts were accessed and examined.

## 3. Results

**Figure 1:**
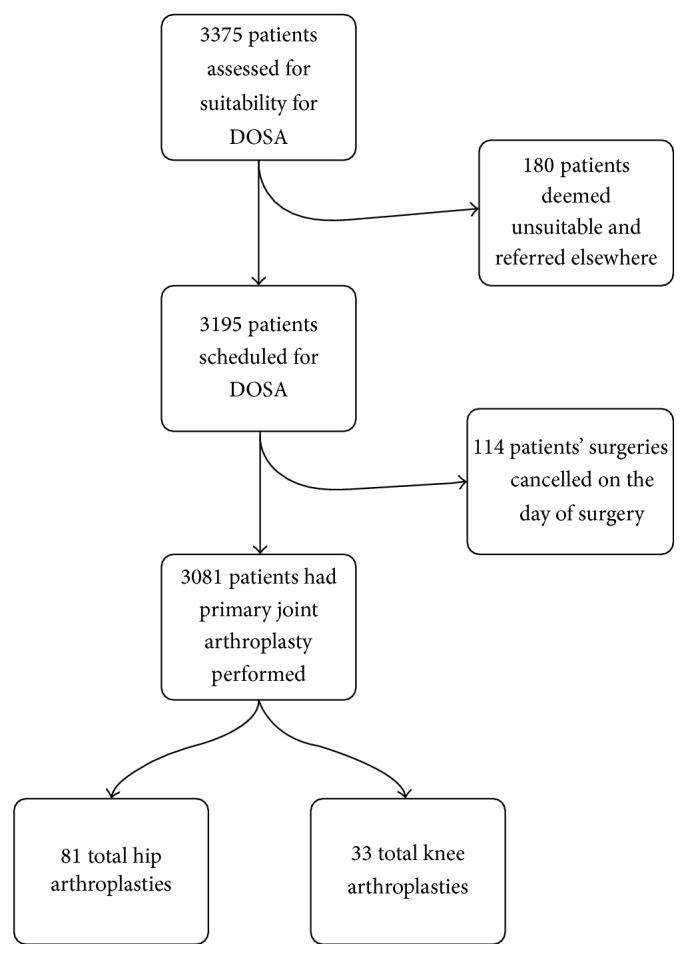
Total patients assessed over the study period.

One hundred and fourteen (3.5%) patients' surgeries were cancelled on the day of the surgery (Figure 1). Of the patients whose surgeries were cancelled on the day of the proposed surgery, the mean age was 73 years (range 18–93). There were 51 male and 63 female patients' surgeries cancelled. Patient demographics and ASA grades are shown in Table 1.

Ninety-two patients' surgeries were cancelled for medical reasons. The remaining 12 patients' surgeries were cancelled for other reasons, namely, patient decision (*n* = 7), attending surgeon being ill (*n* = 4), and technical issues with theatre (*n* = 1). One patient was a Jehovah's Witness and refused blood transfusion. He was recategorized into high risk on this basis.

The most common medical reason for cancellation was cardiac related illness (*n* = 27). Nineteen of these patients had preexisting cardiac conditions. Decompensated CCF was the most common cardiac reason for a patient's surgery to be cancelled. Other cardiac related reasons included uncontrolled hypertension, unacceptably high risk from coronary artery disease, and non-rate controlled atrial fibrillation. 15 of the patients in the study were sent to have an echocardiogram prior to being deemed fit for surgery. Nine patients were referred to cardiology subsequently to initial cancellation.

22 patients' surgeries were cancelled due to respiratory disease. Six of these patients were smokers and four were ex-smokers. Six patients had a previous diagnosis of COPD. Most of these cancellations were due to exacerbations of COPD or lower respiratory tract infections.

The miscellaneous reasons for cancellations are demonstrated in Table 2. Overall 57 (50%) patients' surgeries cancelled were due to chronic medical conditions, which were present at the time of PAC.

Three patients had their operation cancelled on more than one occasion. 77 patients (67.5%) had their operation rescheduled and performed in our institution at a later date. The median length of stay of these patients was eight days, which is one day longer than the overall median length of stay of 7 days.

## 4. Discussion and Conclusion

Previous studies have demonstrated patient satisfaction with DOSA and the cost benefit has been repeatedly proven [8, 11, 12]. Cancellation on the day of surgery not only is expensive but can be emotionally upsetting for patients and their families [13]. Although some cancellations are unavoidable, preoperative anesthetic assessment is effective in reducing the risk of perioperative complications after joint arthroplasty and is vital to the effective function of DOSA [14, 15].

The aim of the current study was to evaluate the success of DOSA in a regional specialty orthopedic unit and identify reasons for patient surgery cancellation. To the best of our knowledge this is the first paper to specifically analyze DOSA cancellations for lower limb arthroplasty.

### 4.1. Limitations

Our study has a number of limitations. (1) This is a retrospective study and patient records may not be complete. (2) There is often undocumented communication between the PAC, anesthesiologist, and medical physicians, which may not have been recorded in the patient notes. (3) There are many conditions such as LRTI and UTIs and exacerbations of CCF that are not precisely quantified either in PAC or in the day of surgery note. (4) Patients may have undergone the cancelled procedure at a later date in another institution without this information being available.

### 4.2. Comparison to Previously Published Results

In the current study 3195 patients were deemed fit for surgery and scheduled for admission on the day of their procedure. 114 (3.5%) of these patients' surgeries were cancelled on the scheduled day of surgery, representing one cancellation approximately every thirteen operating days. This rate compares well to DOSA cancellations rates reported in the literature for all surgical specialties which range from 4.6 to 13.2% [8]. A Finnish group reported a rate of 5.4% for a subset of elective orthopedic procedures in their cohort [8]. Mangan et al. had a cancellation rate of 10% of patients admitted for joint replacement surgery, although not all of these patients underwent preoperative assessment [16]. Our unit has the advantage of being a stand-alone specialty orthopedic unit, thereby eliminating surgical cancellations due to bed unavailability and surgical emergencies diverting staff. Conversely, there is no access to intensive care facilities and no possibility of same day specialty medical review.

### 4.3. Cardiac Considerations

Ninety-two patients' (82%) surgeries were cancelled as the anesthesiologist felt they represented an unacceptable medical risk or medical comorbidities had not been sufficiently optimized. Cardiac comorbidities were the commonest reason for day of surgery cancellations (*n* = 27), with decompensated congestive cardiac failure (CCF) representing the reason for cancelation in the greatest portion of these patients. Other cardiac conditions leading to cancellation include uncontrolled hypertension, tachycardic arrhythmia, and anesthetic concerns relating to preexisting ischemic cardiac disease.

Cardiac related illness is a major consideration for patients undergoing elective surgical procedures and second only to surgical site infection as a reason for readmission following surgery [17]. There have been clear guidelines related to evaluation of ischemic heart disease, CCF, and valvular heart disease, produced both in Europe (European Society of Cardiology) and in the USA (American College of Cardiology/American Heart Association (ACC/AHA)), that clarify many of the considerations [18, 19]. They identify conditions that need more investigation and possible treatment. At our centre ECG is performed in all patients above 40 years of age as recommended by all guidelines [18–20]. Stress testing is recommended for investigating possible coronary artery disease in patients with risk factors [19]. Dobutamine stress echocardiograph is an alternative in our relatively immobile cohort [19]. It is effective in identifying patients without a history of ischemic heart disease who are at high risk of cardiac morbidity based on risk factors [21]. A left ventricular ejection fraction of less than 35% is associated with a worse prognosis in vascular surgery but its relevance to orthopedic surgery has not been proven [19, 22]. The European Society for Cardiology guidelines reference the use of brain natriuretic peptide (BNP), a marker of heart failure, as an investigation to be performed in some instances but there is no enough evidence to recommend it as an appropriate investigation [19]. Conditions that specifically need to be addressed prior to surgery are summarized as follows.

*Cardiac Conditions Requiring Mandatory Investigation*

- Unstable coronary syndromes
  1. Unstable or severe angina
  2. Recent myocardial infarction (within 4–6 weeks)
- Decompensated heart failure
  1. Inability to carry out any physical activity without discomfort
  2. Symptoms of cardiac insufficiency at rest, such as fatigue, palpitation, or dyspnea
  3. Discomfort that is increased with physical activity
  4. Worsening or new-onset heart failure
- Substantial arrhythmias
  1. High-grade, Mobitz type II or tertiary atrioventricular block
  2. Symptomatic ventricular arrhythmias
  3. Supraventricular arrhythmias (including atrial fibrillation) with heart rate of >100 beats/min at rest
  4. Symptomatic bradycardia
  5. Newly recognized ventricular tachycardia
- Severe valvular disease
  1. Severe or symptomatic aortic stenosis
  2. Symptomatic mitral stenosis (progressive dyspnea on exertion, exertional presyncope, and heart failure)

The published guidelines do not dictate the institutional requirements or level of medical care necessary for such patients undergoing elective orthopedic surgery. There is no intensive care unit on-site or anesthesiologist on call at our institution, which is not uncommon for a “stand-alone” specialty surgical centre. Most of the published guidelines are focused on risk stratification rather than setting out clear conditions that must be satisfied for the operation to proceed. Ideally all patients with any cardiac issue would see a cardiologist prior to surgery but this may not always be feasible. The ACC/AHA recommend open communication between the PAC/anesthesiologist and the cardiologist rather than a formal consultation in all cases. If a formal consultation is requested specific questions should be asked regarding the perioperative management [18]. The PAC and anesthesiologist need to be aware of the indicated investigations so that reasonable requests will be made and resources will not become saturated. Conditions requiring mandatory investigation are summarized in “*Cardiac Conditions Requiring Mandatory Investigation*” [23].

One patient's surgery was cancelled due to lower limb pitting oedema. In cases of CCF lower limb oedema is not seen as a reliable sign and an elevated jugular venous pressure and positive hepatojugular reflux are more reliable as indicators of hypervolaemia [24, 25]. Thirty-six percent of the patients (*n* = 41) with cancelled surgery in our institution were on treatment for hypertension. Mild or moderate hypertension is not an independent risk factor for perioperative cardiovascular complications. Bozic et al. reported hypertension as the most prevalent comorbidity encountered at preoperative assessment of patients undergoing THA and it is present in 66% of the population in their study [26].

### 4.4. Respiratory Disease and Smoking

The second commonest reason for cancellation of surgery was respiratory comorbidity. Preoperatively, a focused medical history, respiratory exam, and chest X-ray were standard for all patients over 70 years of age and those with preexisting respiratory disease. Quantifying respiratory risk was at the PAC's/anesthesiologists' discretion. Risk factor stratification indices have been developed to identify patients at risk of postoperative respiratory failure and pneumonia [27–30]. The introduction of these assessment tools may allow for improved concordance between the PAC and the anesthesiologists' respiratory evaluations.

Smoking status is included in risk factor indices as a risk for both pulmonary and cardiac disease [28, 30]. Smoking cessation for as little as two weeks has been shown to reduce risk of complications [31]. In orthopedic surgery, higher rates of infection and osteomyelitis and poor functional outcome scores have been demonstrated in smokers. A cessation period of 4 weeks has reduced the levels of these poor outcomes and brought them closer to that of the general population [32]. An introduction of a smoking cessation officer at our institution may help to reduce both cancellations and complications.

Patients with a history of chronic obstructive pulmonary disease, a disease usually associated with smoking, have odds ratio of 4.2–4.5 for developing respiratory failure after major surgery [30, 33]. It is therefore imperative that conditions of these patients are fully optimized before undergoing major surgery. Most exacerbations are infective and related to the severity of COPD and smoking status [34]. Those at the highest risk of exacerbations may need to be assessed more regularly and closer to the time of surgery.

### 4.5. General Considerations

With all arthroplasty procedures there is a level of risk. Older patients and those with comorbidities are at higher risk of complications [35]. The increased risk associated with comorbidity is not limited to the perioperative period; those with higher comorbidity indices are at higher risk of reoperation in the first 2 years [36]. Once conditions have been optimized the decision is shared between the surgeon, the anaesthetist, and the patient as to whether this risk is acceptable and they are happy to proceed with arthroplasty. Six patients (5% of total) in our study cancelled their own procedure on the day of surgery. Caesar et al. had a much higher percentage of patient choice cancellation (33%) although their study included patients scheduled for surgery but who cancelled prior to admission. Careful counseling and scheduling of patients in the office may account for our relatively low rate of voluntary withdrawal from surgery [15]. Risks should be made clear to the patient well in advance of admission so that if they decide to forego the procedure another patient can be scheduled. Further brief correspondence with the unit within a week of surgery is effective in reducing cancellations and this process is in place at our institution [37].

In the current study, the cancellation rate of 3.5% is well below the aspirational national HSE target rate of 5% [9]. While a 0% cancellation rate is unrealistic, there was relatively large proportion of the patients surgeries cancelled secondary to chronic medical conditions. These patients should clearly have been identified at PAC, their condition optimized, and those patients who were unsuitable for procedure in a stand-alone unit transferred to a more suitable location. We feel that there is a need for development of protocols at a local level by the physicians involved in running the PAC and the anesthesiologist caring for the patient on the day of surgery. Discrepancies between what the anesthesiologist and what the PAC physicians deem safe for patients being admitted on the day of surgery is clearly a major factor in cancellation. Communication including all medical professionals involved in the provision of the patient's care has previously been cited as a vital ingredient in preoperative evaluation [38].

In conclusion day of surgery admission is associated with a low rate of cancellations on the day of surgery. Patients with cardiorespiratory comorbidities are at the greatest risk of cancellation. We recommend such patients undergo more intensive preoperative investigation and suggest development of tighter protocols between medical and anesthetic personnel regarding cancellation of surgery.

## Figures and Tables

**Table 1:** Patient demographics, ASA grade, and smoking status.

Patient characteristics	
Sex (M : F)	51 : 63
Age	73 (18–93)
ASA grade	2.38
Time from preassessment	56 (2–579) days

Scheduled for hip arthroplasty	81
Scheduled for knee arthroplasty	33

Smoking status	
Yes	15
No	48
Ex-smoker	17
Not recorded	24

**Table 2:** Reason for Cancellations.

Reason for Cancellation	Number of Patients
Cardiac	27
Respiratory	22
Haematology	7
Renal	1
URTI	3
MRSA	2
AAA	1
Soft Tissue	9
Patient	7
Urological	6
Cancer	1
Vertigo	1
Fracture	1
Liver Disease	1
Medication	5
Neurological	2
Surgical	3
Technical	6
Unspecified Medical Reason	9
